# Antimicrobial Activity of Green Synthesized Silver Nanoparticles Using Waste Leaves of *Hyphaene thebaica* (Doum Palm)

**DOI:** 10.3390/microorganisms11030807

**Published:** 2023-03-22

**Authors:** Nadiyah M. Alabdallah, Essam Kotb

**Affiliations:** 1 Basic and Applied Scientific Research Center (BASRC), Imam Abdulrahman Bin Faisal University (IAU), P.O. Box 1982, Dammam 31441, Saudi Arabia; 2 Department of Biology, College of Science, Imam Abdulrahman Bin Faisal University (IAU), P.O. Box 1982, Dammam 31441, Saudi Arabia

**Keywords:** silver nanoparticles, antibacterial, anticandidal, characterization, doum palm

## Abstract

Silver nanoparticles (AgNPs) were biosynthesized for the first time from waste leaves extract of local doum palms in Tabuk, Saudi Arabia. The transmission electron microscope (TEM) revealed a spherical shape with a particle size from 18 to 33 nm. The d-spacing is about 2.6 Å, which confirms a face-centered cubic crystalline building. The biosynthesized AgNPs were evaluated as an antimicrobial agent against several pathogenic bacteria, including *Escherichia coli* ATCC 25922, *Staphylococcus aureus* ATCC 29213, and *Pseudomonas aeruginosa* ATCC 27853. The highest action was exerted against *S. aureus* ATCC 29213 (MIC = 1.5 µg/mL). Interestingly, AgNPs also showed anticandidal activity against the pathogenic yeasts *Candida albicans* ATCC 14053 (MIC = 24 µg/mL) and *Candida tropicalis* ATCC 13803 (MIC = 96 µg/mL). Scanning electron microscope (SEM) revealed deep morphological changes in *Candida* spp. due to the treatment of the AgNPs. Scarce pseudohyphae, perforation, exterior roughness, irregularly shaped cells, and production of protective exopolysaccharide (EPS) were the main features. In conclusion, the process of biosynthesis of AgNPs from the aqueous leaf extract of *Hyphaene thebaica* is environmentally compatible and induces the biosynthesis of tiny AgNPs that could be a promising candidate in biomedical applications, including antimicrobials against some pathogenic bacteria and yeasts.

## 1. Introduction

Multidrug resistance is a huge health concern worldwide as it results in high mortality and morbidity. This dilemma resulted from the overuse and misuse of antibiotics in various medical and non-medical applications. The resulting drug-resistant strains can cause serious infections of high morbidity [1]. For instance, the Gram-positive bacterium *S. aureus* evolved methicillin-resistant strains called MRSA strains; these also became resistant to all known β-lactam antibiotics, including cephalosporins, penicillins, and carbapenems [2]. Other strains of MRSA became unaffected by exposure to vancomycin, linezolid, and daptomycin, making *S. aureus* a leading cause of death worldwide. These resistant strains can produce severe diseases such as endocarditis, osteomyelitis, pneumonia, and sepsis if they reach the circulatory system [2]. In the case of Gram-negative bacteria, such as *P. aeruginosa*, it is an opportunistic pathogen causing a high level of morbidity in cystic fibrosis and immunocompromised people. Control of *P. aeruginosa* is very challenging because of its remarkable ability to neglect the action antibiotics due to its biofilm-mediated resistance, high level of intrinsic resistance, and acquired resistance mechanisms [3]. The pathogenic bacterium *E. coli* has two major contributors to its intrinsic resistance against antibiotics. The first contributor is its impermeable outer membrane. The second contributor is its ability to express many efflux pumps that successfully lower the accumulation of drugs intracellularly [4]. According to surveillance data from the centers for disease control and prevention (CDC), antifungal resistance of pathogenic *Candida* is also an increasing problem over the past 20 years. *Candida* infections may resist antifungal drugs such as fluconazole and echinocandins, making them difficult to treat, causing severe infections in our mouth, skin, vagina, and life-threatening circulatory diseases. The most common human pathogen belonging to *Candida* is *C. albicans* [5].

Relatively recently, nanotechnology attracted significant attention in the field of antimicrobials and other applications in medicine and the environment. The secret behind their distinctive physicochemical and biological properties relies on their nano size, which gives them a high surface-to-volume ratio [6]. Contrary to antibiotics, nanoparticles can escape the counteraction of bacterial cells due to exposure by breaking the bacterial resistance mechanisms such as β-lactamases, the expression of efflux pumps, impermeability of the outer membrane, and the modification of the target site or target process [6]. In addition, nanoparticles use different antimicrobial actions comprising interruption of cell membrane consistency and induction of reactive oxygen species (ROS) production, which inhibits RNA synthesis and protein synthesis [6]. Nanoparticles prepared from noble metals such as silver have been broadly studied for biological applications and other concerns. The significance of AgNPs as antimicrobial agents is superior to other noble metals due to their antimicrobial properties, fast healing of wounds, and additional applications [7]. Due to this, the demand for AgNPs has grown. Estimates showed that around 320 tons of AgNPs are made every year around the world by chemical, physical and biological methods [6,7].

Nanoparticles can be produced utilizing top-down and bottom-up techniques. Top-down methods comprise bulk metal reduction with advanced ablations such as sputtering, mechanical grinding, thermal decomposition and green synthesis techniques. In contrast, the bottom-up procedures involve constructing nanoparticles atom by atom [8]. The chemical and physical methods are effective in the production of nanoparticles; however, the chemical methods may include toxic chemicals as stabilizing agents, which may cause toxicity upon use [7]. In addition, their biocompatibility is very low and possesses latent biological risks. Further steps may be needed to avoid particle agglomeration. In addition, they are hazardous upon leakage into the environment. The physical methods, on the other hand, expend much energy, are expensive and give low yields [9].

Recently, the green synthesis of metal nanoparticles has arisen, which is centered on the utilization of plant extracts as bioreductants. This approach provides safer, easier, faster, more eco-friendly and economical nanoparticles when compared with the physical and chemical approaches and safe alternatives for antimicrobial applications [7,10]. Successful biosynthesis of AgNPs from many plant extracts has been carried out by many authors. An effort was made using *H. thebaica* fruit extracts. *H. thebaica* is traditionally recognized as a gingerbread tree or doum tree in the Arab region [10]. It is one of the most important medicinal desert plants. It contains high amounts of polyphenols, minerals, vitamins, saponins, tannins, essential oils, linoleic acid, coumarins, flavonoids, hydroxycinnamic acids, carbohydrates and fibers. These have very important health benefits, including the treatment of hematuria, bleeding, dyslipidemia, as an antihypertensive, diaphoretic, diuretic, or to lower blood pressure [9,10]. The extract of doum fruits exhibited antimicrobial and antioxidant properties in many studies. Although the biological activities of doum fruits have been investigated in different studies, other parts, such as leaves, have received less attention from researchers [7,8,9]. This research aimed at the biosynthesis of small-sized AgNPs from the aqueous waste leaf extract of *H. thebaica* for the first time and explored both their antibacterial and anticandidal activity against some pathogenic strains of bacteria and yeasts.

## 2. Materials and Methods

### 2.1. Plant Material, Microbial Strains, Media, and Reagents

Waste plant leaves were gathered from a local garden in Tabuk, Saudi Arabia, and washed with distilled water thoroughly three times to remove any dust and undesirable surface particles. The ATCC microbial strains were helpfully gifted from King Fahd University Hospital, Khobar, Saudi Arabia. Silver nitrate ACS reagent, ≥99.0% purity, Mueller-Hinton agar (MHA) and yeast extract peptone dextrose (YPD) broth were purchased from Sigma Aldrich. Other chemicals were obtained from local suppliers in the analytical grade form.

### 2.2. Green Synthesis of AgNPs

AgNPs were fabricated greenly using waste leaves extracted from the doum palm [*H. thebaica* (L.) Mart.] as capping, stabilizing, reducing agents and silver nitrate following the methods of Ahmed et al. [11] and Lima et al. [12]. The waste leaves were collected and thoroughly washed with distilled water. Then 10 g of sundried powder was mixed with 100 mL of deionized water in a 500 mL beaker and incubated in a shaking incubator at 35 °C for 3 h at a speed of 100 rpm. Thereafter, it was filtrated using Whatman filter paper 11.0 µm. Next, 100 mL of 10 µM AgNO_3_ solution was blended with 50 mL of the aqueous extract from the leaves of the *H. thebaica* (L.) Mart. at 2:1 ratio (*v*/*v*) and incubated at 40 °C until the color changed to brown.

### 2.3. Characterization of AgNPs

Several analytical techniques were used to analyze the properties of AgNPs. The UV–visible spectroscopy method was utilized in order to carry out the beginnings of the characterization process for the AgNPs. The UV–Vis spectrometer from Shimadzu-UV 1800 was used to measure the absorbance of the colloidal sample in the range of 200–800 nm, with distilled water as a comparison. The FTIR RX1-Perkin Elmer was used to analyze the functional group responsible for the silver nanoparticles in the wavelength range of 5000–100 cm^−1^. The TEM was applied to determine the exact morphology and particle size of the biosynthesized AgNPs.

### 2.4. In-Vitro Antimicrobial Activity of AgNPs

The biosynthesized nanoparticles stock suspension was set up at a dilution of 128 µg/mL sterile Dimethyl sulfoxide (DMSO). It was then homogenized by sonication for 30 min. The initial antimicrobial effect of the fabricated sample was assessed by the measurement of formed inhibition zones (mm) in MHA according to the standard Beecher and Wong [13] method. Seeded clinical microbial strains were *P. aeruginosa* ATCC 27853, *E. coli* ATCC 25922, *S. aureus* ATCC 29213, *C. albicans* ATCC 14053 and *C. tropicalis* ATCC 13803. The MHA was adjusted to pH 7.0 and then sterilized by autoclaving for 20 min at 121 °C. After settling the medium in Petri dishes, the selected bacteria and yeasts at 1.0 × 10^8^ CFU/mL were swabbed on the surface of an agar layer. Thereafter, filter paper disks of 5 mm diameter were positioned onto the agar surface. The target AgNPs were then applied at 5 µL volume per disk. Incubation of the cultures was carried out for 24 h at 37 °C. The width of the clearing zones was evaluated to verify the antimicrobial potential of AgNPs prepared from doum palm [14]. 

For minimal inhibitory concentration (MIC) determination, the broth dilution method was adopted [15]. The initial dilution was adjusted to 128 µg/mL in a total volume of 4 mL of sterile Brain Heart Infusion Broth (BHIB) in test tubes. Two-fold dilution series were then made to achieve 64 µg/mL, 32 µg/mL, 16 µg/mL, 8 µg/mL, 4 µg/mL, 2 µg/mL and 1 µg/mL dilutions. Two hundred microliters of bacterial and/or yeast suspensions at 1.0 × 10^8^ CFU/mL were introduced in separate tubes. Tubes not containing NPs served as positive controls (100% microbial growth), whereas tubes without microbial inoculations served as negative controls (0% microbial growth). Incubation was carried out for 24 h at 37 °C under static conditions. After incubation, 440 µL filter-sterilized resazurin was added, and the tubes were further incubated overnight under the previous condition. MIC was defined as the average of the lowest concentration of AgNPs inhibiting the growth and the highest concentration of AgNPs allowing the growth of tested microbes [15].

For minimum bactericidal concentration (MBC) and/or minimum fungicidal concentration MFC determination, 100 µL from the tube contents above the value of MIC were plated on the surface of MHA. At the MBC values, no colony growth was observed. 

All microbial nutrient media in this study were made following the producer’s standards. Microbial strains were preserved in Heart Infusion Agar (HIA) at 4 °C and subcultured on a nutrient medium for 24 h at 37 °C before proceeding with the antimicrobial assessments.

### 2.5. Tolerance Level

The tolerance of the tested microbes against AgNPs was assessed according to Mondal et al. [15] following the formula: Tolerance = MBC (or MFC)/MIC. When the tolerance is equal to or more than 16, it indicates a static effect of NPs, whereas it is considered a cidal effect when it is equal to or less than 4.

### 2.6. Anticandidal Activity under SEM

The exterior and topology of the fungal cells of both *C. albicans* ATCC 14053 and *C. tropicalis* ATCC 13803 after treatment with AgNPs were examined with the help of SEM. Initially, YPD broth was formulated from yeast extract (10 g/L, *w*/*v*), peptone (20 g/L, *w*/*v*) and dextrose sugar (20 g/L, *w*/*v*) at pH 6.5. The medium was sterilized for 15 min at 15 lbs pressure. Two loopfuls of yeast cells were used to inoculate a 25 mL medium. Treated cultures were exposed to a AgNP suspension at 5 µg/mL concentration. Blanks were prepared by replacing the AgNP suspension with distilled deionized water. Incubation was done at 37 °C at a speed of 180 rpm for 24 h to allow cell growth.

For the processing of cell examination under SEM, cells were harvested by centrifugation at 5000 rpm for 15 min. Cells were then fixed with 10% formaldehyde for 24 h. Dehydration of cells was carried out in ascending concentrations of ethanol (10%, 30%, 50%, 70%, 90% and absolute ethanol). Exposure time to alcohol was set to 10 min except for the absolute ethanol treatment, which was left for 1 h. Drying was then performed at 35 °C for 24 h. The examination was performed using VEGA3 TESCAN SEM.

### 2.7. Statistical Analysis

All the measurements and treatments were performed in triplicates unless otherwise stated. The statistical analysis was performed using the SPSS Statistics V24 software. The final readings were represented in the form of averages ± standard deviations.

## 3. Results

### 3.1. Characterization of AgNPs

**Figure 1:**
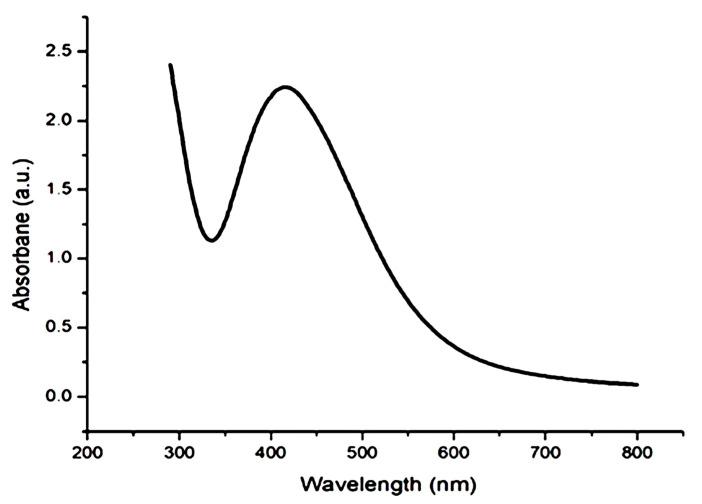
The UV spectrum of the greenly synthesized silver nanoparticles.

AgNPs in the current research were fabricated by mixing the leaf extracts of *H. thebaica* (L.) Mart with AgNO_3_ at 40 °C until a brown color was obtained. In UV–visible spectroscopy analysis, silver nanostructures were suspended in deionized water and revealed in the 200–800 nm range. The UV–visible investigation showed a strong peak at 400 nm (Figure 1).

**Figure 2:**
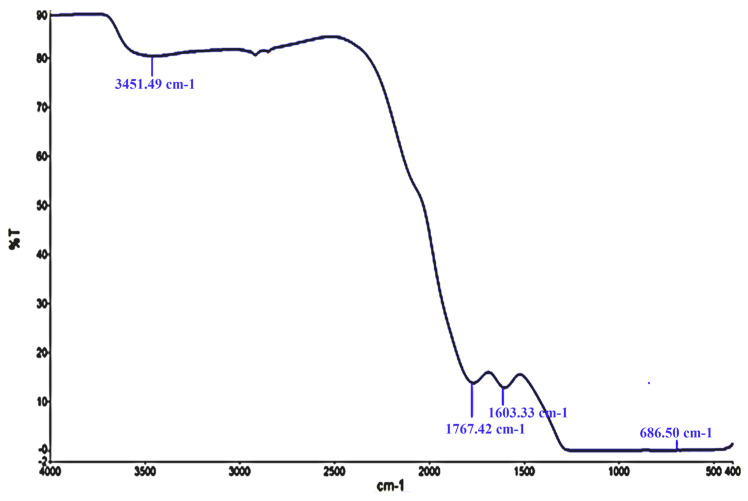
The FTIR pattern of biosynthesized silver nanoparticles from the aqueous leaf extract of *H. thebaica*.

Next, FTIR spectroscopy was employed to determine the functional groups presenting the synthesized silver nanoparticles. The FTIR pattern showed strong IR bands at 3451, 1767, 1603 and 686 cm^−1^ (Figure 2).

**Figure 3:**
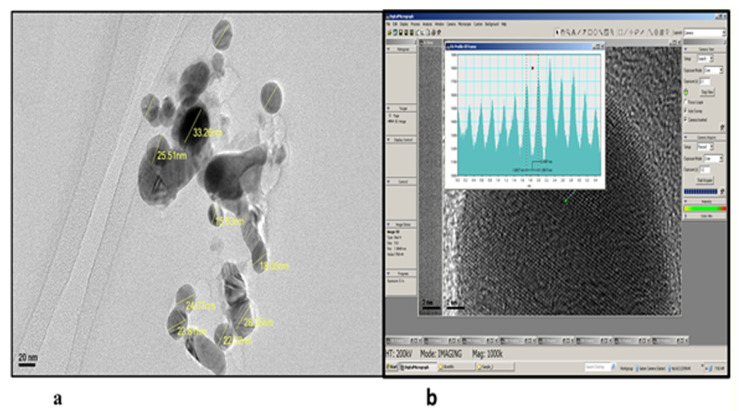
The TEM investigation (**a**) the particle size of silver nanoparticles (**b**) the AgNPs d-spacing was 2.6 Å, with a crystalline structure.

The TEM investigation has demonstrated that AgNPs prepared by the *H. thebaica* leaf extract were spherical with a dimension of 18–33 nm (Figure 3a). The distance between fringes (d-spacing) was about 2.6 Å, which confirms a face-centered cubic crystalline structure (Figure 3b).

### 3.2. Antimicrobial Study

**Figure 4:**
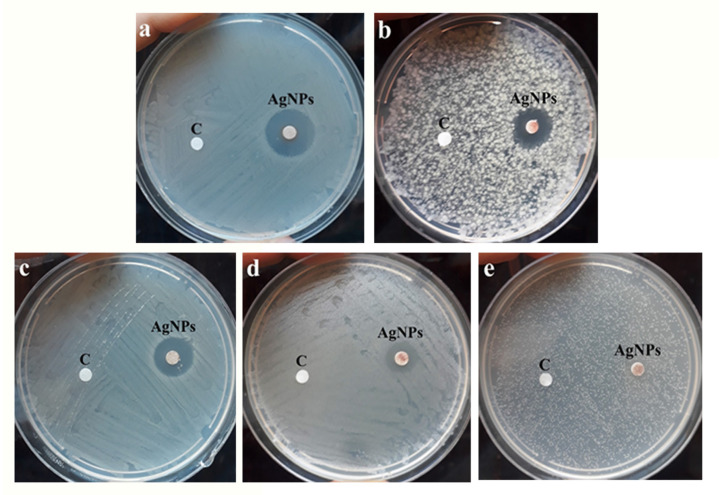
The antimicrobial activity of the newly prepared AgNPs against cultures of different pathogenic microbes. The inhibition zones in mm were measured after 24 h of incubation at 37 °C. Filter paper disks of 5 mm were positioned on the surface of Mueller Hinton agar then 5 µL of 128 µg/mL AgNPs prepared from doum palm waste leaves were applied onto each disk. (**a**) represents *S. aureus* ATCC 29213, (**b**) represents *P. aeruginosa* ATCC 27853, (**c**) represents *E. coli* ATCC 25922, (**d**) represents *C. albicans* ATCC 14053, whereas (**e**) represents *C. tropicalis* ATCC 13803. Disk C corresponds to the control treatment (solvent alone).

From Table 1 and Figure 4, it appears that AgNPs have activity against all the tested microorganisms. The MICs ranged from 1.5 to 96 µg/mL. The highest action was exerted against the Gram-positive bacterium *S. aureus* ATCC 29213 (MIC = 1.5 µg/mL). While AgNPs have exerted lower activity against the Gram-negative bacteria *E. coli* ATCC 25922 (MIC = 3.0 µg/mL) and *P. aeruginosa* ATCC 27853 (MIC = 6.0 µg/mL). Interestingly, the NPs have exerted antifungal activity against the unicellular pathogenic fungi *C. albicans* ATCC 14053 (MIC = 24 µg/mL) and *C. tropicalis* ATCC 13803 (MIC = 96 µg/mL). The results of the MIC experiment coincided with those observed with the inhibition diameters in Figure 4.

Furthermore, the values of MBCs of the greenly synthesized AgNPs were successfully evaluated against the five microorganisms. The values ranged from 4 to 256 µg/mL. The best MBC value was obtained against the Gram-positive bacterium *S. aureus* ATCC 29213 (4 µg/mL), whereas the lowest values were obtained against Gram-negative bacteria and pathogenic yeasts.

**Figure 5:**
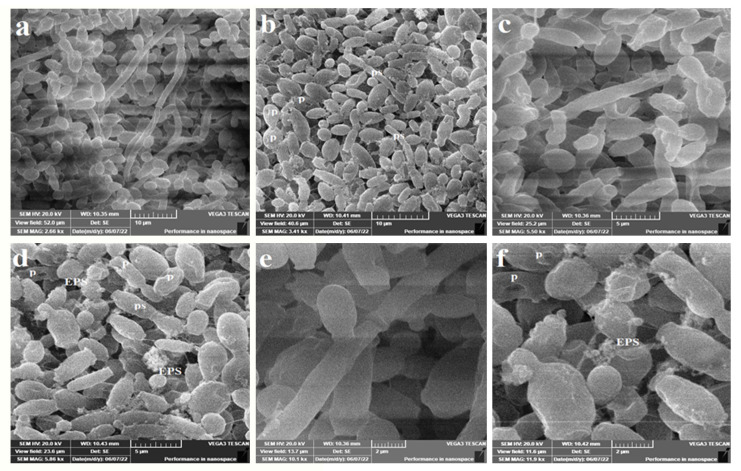
The SEM micrographs of *C. tropicalis* ATCC 13803. (**b**,**d**,**f**) represent treated cells with AgNPs at 5 µg/mL concentration in YPD broth for 24 h. (**a**,**c**,**e**) represent the untreated cells. Treated cells show reduced pseudohyphae (ps), perforation (p), roughened irregularly shaped cells and exopolysaccharide (EPS) production.

The ultrastructural differences under SEM revealed that *C. tropicalis* exposed to AgNPs at 5 µg/mL concentration suffered scarce pseudohyphae (Figure 5b,d,f), perforation (Figure 5b,d,f), exterior roughness (Figure 5b,d,f), irregularly shaped cells (Figure 5f) and EPS formation (Figure 5f). In parallel, untreated cells have shown well-established clusters with prominent pseudohyphae along with the dominant yeast cells (Figure 5a,c,e). The produced EPS from *C. tropicalis* is a means of cell protection against conventional antifungals and may also be a reason for the diminished susceptibility of cells against AgNPs (Figure 5f). In addition, this may be the reason for the high MIC when compared with *C. albicans* (96 µg/mL vs. 24 µg/mL, Table 1).

**Figure 6:**
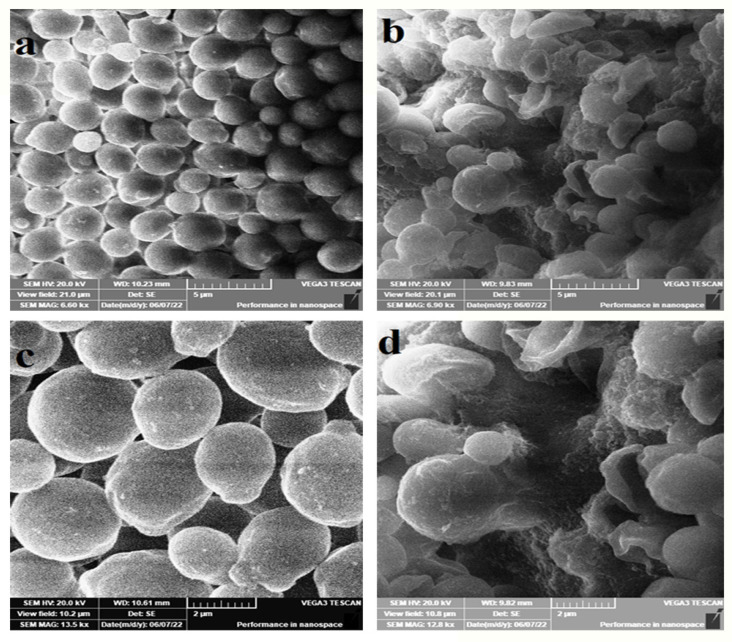
The SEM micrographs of *C. albicans* ATCC 14053. Panels b and d represent treated cells with AgNPs at 5 µg/mL concentration in YPD broth for 24 h. Panels a and c represent the untreated cells. Treated cells show intensive cell damage, deformation and irregularly shaped cells (**b**,**d**) when compared with untreated cells showing well-established cell clusters (**a**,**c**).

In the case of *C. albicans*, SEM revealed that AgNPs induced intensive cell damage, deformation, and irregularly shaped cells (Figure 6b,d) when compared with untreated cells showing well-established cell clusters (Figure 6a,c).

## 4. Discussion

The formation of AgNPs with the help of plant extracts was completed in many studies to inhibit the growth of pathogenic microorganisms, the progression of cancer cells as well as other applications. Their biological activity was found to differ with their shape, size and the plant part used. The antibacterial application of AgNPs was tested for many of the prepared AgNPs, whereas the anticandidal activity was studied in a few reports. The present NPs were found to exert a prominent antimicrobial efficiency against five of the most common pathogenic bacteria and *Candida* spp.

Doum palm trees are common in the Arab region and were selected in this study. The production of AgNPs by the waste leaves of doum palm extract was confirmed by the color change of the reaction mixture towards orange to brown colors, which was an indication of the reduction in Ag^+^ ions to Ag° [16,17]. This color change is the initial, observable sign of AgNP production because the excitation of the AgNP’s surface plasmon induces a color shift in solutions [18]. The production of AgNPs from AgNO_3_ is verified by several analytical protocols such as FTIR spectroscopy, UV–visible spectrophotometry and TEM. UV–visible spectroscopy is an applicable technique that verifies the biosynthesis of AgNPs [19]. In accordance with our result, Singh et al. [20] also stated that the UV spectra of silver nanoparticles were maximal at the wavelength of 400 nm.

In the FTIR analysis, the band at 3451 is attributed to the O–H mode vibration, band 1767 corresponds to carbonyl stretching vibrations, band 1603 shows N–H band primary amines in a protein, and the band at 686 is assigned to the O–H vibration mode. These results collectively indicate that these AgNPs were surrounded by phytochemical compounds, which reduced the metal. The O-H, N-H and C=O groups were adsorbed on the AgNP’s surface and engaged in the bioreduction of AgNO_3_. In parallel, the FTIR spectrum of the formed AgNPs with the help of *H. thebaica* fruit extract. Mohamed et al. [9] revealed vibrational bands of =C-H, -C-O-C, -C-O, C=O, O=C=O and C-H, indicating that the synthesized nanoparticles were adsorbed by plant components such as proteins, phenols, flavonoids, terpenoids, organic acids and other active constituents. The findings of the FTIR study have generally recognized the existence of carbonyl groups from the amino acid residues of plants, therefore, indicating the role played by plant proteins and other phytochemicals in the biosynthesis of AgNPs. It is difficult to figure out the role of a specific phytochemical [21]. Referring to the literature, AgNP preparation from *H. thebaica* fruit extract gave larger particle sizes reaching 70 nm [9]. In addition, the preparation of AgNPs from *Origanum vulgare* aqueous leaf extract gave a particle size of 136 nm. For this, their antimicrobial potential was lower when compared with the current nanoparticles because the efficacy of AgNPs was found to be dependent on physical characteristics, such as size and shape. The smaller the size of the AgNP, the better its ability to enter the cell through the cell wall and cell membrane, hindering the structural molecules and the critical metabolic pathways at the cellular and subcellular level, therefore inhibiting the bacterial growth, cell division and eventually can cause cell death [22]. Regarding other studies, the preparation of AgNPs from *H. thebaica* was carried out in one paper using fruit extract [9]. In this research, we used the leaf extract of *H. thebaica* for the first time. This gave a smaller size and more potent nanoparticles. AgNPs from *H. thebaica* fruit extract gave particle sizes of 4.5–70 nm with lower inhibition to both bacteria and some molds. The induced nanoparticles from fruit extract did not obstruct the growth of Gram-negative bacteria such as *E. coli* and *K. pneumonia* at concentrations less than 1 mg/mL, whereas the Gram-positive bacteria were more susceptible [9]. 

When compared with AgNPs formed by other plants, a previous study reported the biosynthesis of larger particle sizes (136 ± 10.09 nm) from *Origanum vulgare* aqueous leaf extract. These exerted antibacterial activity against *E. coli*, *Aeromonas hydrophilla*, *Salmonella paratyphi*, and *Shigella dysenteriae* with inhibition halos greater than 10 mm in diameter [23]. Another study biosynthesized AgNPs from the aqueous leaf extract of *Origanum vulgare* with particle sizes from 2 to 25 nm but with some degree of agglomeration that may lower the internalization of individual particles [24]. The biosynthesized AgNPs from the fungus *Phoma exigua* had a particle size of 22 nm [25]. 

The mechanism of action of nanoparticles is not yet clearly understood; some explanations have been proposed. They attach to microbial cells easily because of the negative charge on their cells; subsequently, they penetrate the cell membrane causing structural changes. The membrane permeability and proton motive force are also adversely affected [26]. Other researchers suggest the release of silver ions from AgNPs which interact with thiol groups on the sulfur-containing amino acids leading to the inactivation of enzymes, especially the respiratory chain enzymes generating ROS such as H_2_O_2_ and hydroxyl radicals, which induce oxidative stress in microbes. This results in DNA damage, the depletion of GSH and lipid peroxidation [27], and eventually results in dysfunction and cell death [28]. 

The broad-spectrum activity of AgNPs prepared from doum palm against both Gram-positive bacteria and Gram-negative bacteria (Table 1 and Figure 4) agrees with most previous investigations [29]. The superior activity of the synthesized AgNPs against Gram-positive bacteria may be attributed to the ability of Gram-negative bacteria to express efflux pumps (EPs) in their cell membranes [30,31]. Recently, the AcrAB multidrug efflux system and RND-type efflux pump have been discovered in the Gram-negative bacteria used in this study (*E. coli* ATCC 25922 and *P. aeruginosa* ATCC 27853) [32,33]. In fact, EPs enhance the resistance of Gram-negative bacteria by acting as an export system to a wide range of antimicrobials, including antibiotics and nanoparticles. This mechanism allows Gram-negative bacteria to pump out the antimicrobial agent in less time than it requires for them to be diffused toward the inside of cells; consequently, the intrabacterial concentration becomes less than the effective value [30]. The different potentials of AgNPs against Gram-positives and Gram-negatives may also be attributed to the variation in cell wall composition and width. In Gram-negatives, the cell wall is very rich in lipids, and its thickness is only 3–4 nm compared with the 30 nm thickened cell wall in Gram-positives [34]. 

In order to visualize the association between the tested microorganisms, MIC, MBC, MFC and the tolerance level (ratio MBC/MIC) of AgNPs were evaluated (Table 1). AgNPs could be considered as having strong antimicrobial activity against all the microbial strains, as they scored excellent MIC values (1.5–96.0 µg/mL). In addition, AgNPs showed prominent bactericidal and fungicidal activity (4.0–256 µg/mL). The MBC (or MFC)/MIC ratio was between 1.33–267; it is established in the literature that an antimicrobial compound is considered bactericidal/fungicidal when the ratio of MBC (or MFC)/MIC ≤ 4 [35,36].

The SEM study has revealed ultrastructural changes in both *C. tropicalis* and *C. albicans* exposed when treated with AgNPs. In the case of *C. tropicalis*, scarce pseudohyphae (Figure 5b,d,f), perforation (Figure 5b,d,f), exterior roughness (Figure 5b,d,f), irregularly shaped cells (Figure 5f) and EPS formation (Figure 5f) were the main features. The EPS may be considered a means of cell protection against AgNPs and may also be a reason for the diminished susceptibility of cells to the antimicrobials (Figure 5f). In addition, this may be the reason for the high value of MIC when compared with *C. albicans* (96 µg/mL vs. 24 µg/mL, Table 1). In the case of *C. albicans*, SEM revealed that AgNPs induced intensive cell damage, deformation and irregularly shaped cells (Figure 6b,d). This indicates the ability of AgNPs prepared from doum palm to damage the cell wall and hamper pseudohyphae formation in dimorphic yeasts. This is especially significant as both effects are core virulence tools in the genus *Candida*.

## 5. Conclusions

The extract of the doum palm leaf was successfully used as a reductant in the synthesis of silver nanoparticles. AgNPs showed excellent antibacterial and anticandidal properties against some pathogenic strains of both bacteria and yeasts. The Gram-positive bacterium *S. aureus* ATCC 29213 (MIC = 1.5 µg/mL) showed the highest degree of sensitivity. For this, our study recommends the use of AgNPs as an antimicrobial agent. Much more research on the cyto and systemic toxicities of very small metal NPs (which can concentrate in fat cells, neuronal junctions, muscles, brain, organs and so on) is required before wide-scale application in medicine.

## Figures and Tables

**Table 1:** The antimicrobial activity of the newly prepared AgNPs. The inhibition zones in mm were measured after 24 h of incubation at 37 °C. The minimum inhibitory concentration (MIC), minimum bactericidal concentration (MBC), and minimum fungicidal concentration (MFC) were assessed by the broth dilution method.

Microbe	Antimicrobial Activity			
	Inhibition Zone (mm)	MIC (µg/mL)	MBC (or MFC) (µg/mL)	MBC (or MFC)/MIC
*S. aureus* ATCC 29213	18.0 ± 0.4	1.5	4	2.67
*E. coli* ATCC 25922	14.5 ± 1.0	3	4	1.33
*P. aeruginosa* ATCC 27853	15.0 ± 0.5	6	16	2.67
*C. albicans* ATCC 14053	11.0 ± 0.8	24	32	1.33
*C. tropicalis* ATCC 13803	6.5 ± 0.1	96	256	2.67

## Data Availability

Not applicable.
